# Editorial: Trends in AI4ESG: AI for sustainable finance and ESG technology

**DOI:** 10.3389/frai.2024.1448045

**Published:** 2024-06-25

**Authors:** Peter Schwendner, Jan-Alexander Posth

**Affiliations:** Zurich University of Applied Sciences, School of Management and Law, Institute for Wealth and Asset Management, Winterthur, Switzerland

**Keywords:** ESG, sustainable investing, machine learning (ML), artificial intelligence, Alternative Data, evidence based decision-making

In Finance, there is strong client interest to invest in products that are Environmental, Social and Governance (ESG) relevant.

However, ESG investing is now in a crisis. One reason is a credibility problem as the reality of investment practices often lagged the marketing propositions, often even leading to greenwashing scandals. Second, ESG ratings of different rating agencies vary significantly, making structured evaluations difficult if not impossible. Third, the ambitious political agenda, especially in Europe, wants to reach the “green transformation” of the industry indirectly with financial market regulation, not with direct sectorial industry regulation or an active industry policy. This conflicts with performance-oriented and geostrategic considerations, for example regarding the strategic access to critical raw materials. And finally, there is a fundamental mismatch at the core of ESG investing; while ESG is a long-term concept where benefits might only materialize after one or even two decades, due to a slow change in corporate culture, asset management usually adopts a short-term expectation on stock price (out-)performance. Here, the very mandate and, even more important, the fiduciary duty, of asset managers conflicts with the performance ESG has been able to deliver so far.

All of this has, in recent years, resulted in a markedly disinterest in ESG investments, in a decidedly slump in inflows of AUMs, and, in some cases as, e.g., for U.S. pension funds, in active ESG divestment.

From a theoretical viewpoint, the role of sustainable finance changed from a constraint in the form of ESG exclusion lists to an investment goal. This requires data of much higher quality, detail, and coverage as well as Alternative Data sets. These, for finance new, datasets are large, complex, and unstructured so that it is difficult to analyze them with standard procedures.

From a scientific viewpoint, machine learning and AI offer tools to tackle this data challenge and enable the researcher or analyst to extract meaningful information from these datasets. This information then can help to make evidence-based sustainable finance decisions, resulting in real added investment value.

This Research Topic “*Trends in AI4ESG: AI for sustainable finance and ESG technology*” contains four articles, all of which yield essential insights into how AI and Alternative Data can help to overcome today's challenges in Sustainable Finance:

“*The assessment list for trustworthy artificial intelligence: a review and recommendations*” (Radclyffe et al.).

“*Introducing DynaPTI–constructing a dynamic patent technology indicator using text mining and machine learning*” (Freunek and Niggli).

“*Analyzing global utilization and missed opportunities in debt-for-nature swaps with generative AI*” (Tkachenko et al.).

“*Opportunities for synthetic data in nature and climate finance*” (Tkachenko).

In the first article, Radclyffe et al. review the “seven Principles for Trustworthy AI” proposed by the EU High-Level Expert Group on AI. The authors discuss strengths and weaknesses and present recommendations to both regulators and the industry.

In the second article, Freunek and Niggli present an indicator for patent technology called DynaPTI. The indicator is based on text mining and machine learning. The authors present an application to companies from the wind energy sector.

The third article written by Tkachenko et al. demonstrates using a GPT-4 model to summarize the proliferation of debt-to-nature swaps (DNS) for environmental conservation. They are questioning the efficiency and robustness of DNS against economic and political shocks.

In the last article, Tkachenko reviews the application of synthetic data for nature and climate finance. The author classifies synthetic data production methods and identifies specific data needs of green finance.

## Author contributions

PS: Writing – original draft, Writing – review & editing. JAP: Writing – original draft, Writing – review & editing.

